# Early posterior negativity in humans to pictures of snakes and spiders: effects of proximity

**DOI:** 10.1007/s00221-020-05925-5

**Published:** 2020-10-03

**Authors:** Nick Beligiannis, Jan W. Van Strien

**Affiliations:** Erasmus School of Social and Behavioral Sciences, Brain and Cognition, Erasmus University Rotterdam, PO Box 1738, 3000 DR Rotterdam, The Netherlands

**Keywords:** Early posterior negativity (EPN), Snake detection hypothesis, Proximity, Spatial frequency, Phylogenetic fear

## Abstract

Snakes have proven to drive early attentional capture due to their evolutionary importance, as reflected by the early posterior negativity (EPN). The EPN snake effect might be partly driven by the proximity of the animal. In this study, by employing full-body (medium shot) and head-focused (close-up) pictures, we investigated whether the relative nearness (proximity) of the animal on the picture affects the snake EPN effect. We presented thirty participants with medium shot and close-up snake, spider and bird pictures in a rapid serial presentation paradigm at a presentation rate of three frames per second. We extracted the mean EPN activity from the 225–330 ms time frame after stimulus onset at the parietal–occipital cluster (PO3, O1, Oz, O2, PO4). The results indicate enhanced EPN for snake pictures as compared to spider and bird pictures. In addition, medium-shot snake pictures elicited higher EPN amplitudes than close-up snake pictures, suggesting that the EPN is higher when local, high spatial frequency attributes are visible. Spatial frequency analysis of the stimuli indicated that medium-shot snake pictures possess more power in the high spatial frequency bands, compared to medium-shot spider and bird pictures.

## Introduction

Snakes have been proven to trigger fast detection and early attentional capture in humans and primates, due to their evolutionary importance (Öhman et al. 2001). That is, an evolutionary forced readiness of the visual system seems to be active in the presence of phylogenetic fear (Mühlberger et al. 2006). This suggests a visual attentional mechanism responsible to rapidly detect dangerous stimuli in order to avoid danger and promote survival. The “Snake Detection Theory” (SDT) (Isbell 2006), suggests that snakes, as evolutionary agents, have shaped the development of the primate visual system and have also set vision as the primary sense for threat detection in humans and primates. Previous studies have examined visual attributes of snakes, such as the curvilinear body shape and the snake’s skin patterns, that may play a role in snake detection. The present study was conducted to explore the role of proximity and its role in snake-related attentional capture.

Many studies have acknowledged that there is attentional modulation of the human visual system caused by snakes that is reflected at an electrophysiological level, as measured by event-related potentials (ERPs) (He et al. 2014; Grassini et al. 2016; Van Strien et al. 2014a, b; Van Strien et al. 2014; Van Strien and Isbell 2017). One of the ERPs of primary interest in many studies, is the early posterior negativity (EPN). The EPN is an electrophysiological response occurring predominantly at the parietal–occipital cluster in the 225–300 ms time frame after stimulus onset (Schupp et al. 2006). It has been argued that the EPN is augmented by evolutionary important stimuli (Schupp et al. 2003). These stimuli may activate an evolved fear module in the brain of which the amygdala plays a central role (Mineka and Öhman 2002). The higher EPN amplitudes can be understood as reflecting excitation of the visual cortex through feedforward from the amygdala (e.g., Amaral et al. 2003). One of the paradigms in which the EPN for snake detection has been typically measured is the Rapid Serial Visual Presentation (RSVP) in which stimuli are presented in a randomized manner, at a rate of typically 3 frames per second. The RSVP has been claimed to be an efficient technique to measure attentional processing, as it demands rapid processing of emotional information during intense processing load (Junghöfer et al. 2001).

By employing a RSVP, Van Strien et al. (2014a, b) reported that snake pictures elicited higher EPN amplitudes, compared to spider pictures and bird pictures. This outcome suggests larger activation of the early attentional capture mechanism and the selection of snakes for later processing. In a later study, Van Strien et al. (2014), explored whether the snake-related augmented EPN effect, can be generalized to the entire reptilian class. Interestingly, they reported that snakes exhibited significantly larger EPN amplitudes as compared to other reptiles, therefore further supporting the SDT.

The curvilinear shape, is one of the visual attributes of snakes that may affect rapid snake detection. Van Strien et al. (2016) explored whether the curvilinear shape of the snake is a feature that modulates the EPN. They compared the EPN amplitudes elicited by snakes to the EPN amplitudes of other animals with (e.g., worms) and without curvilinear bodies and reported that snakes were the animals that exhibited the largest EPN component. Interestingly, worms (curvilinear body) elicited higher EPN amplitudes than beetles, suggesting that curvilinear shape cannot be entirely ruled out as a modulating factor in the EPN snake effect.

Another visual attribute that may affect snake detection is partial exposure of the animal; that is, whether the full animal or only parts of it are visible. Van Strien and Isbell (2017) explored whether the snake scale patterns are a driving factor for rapid snake detection in humans. They presented the subjects with a set of close-up snake skin, lizard skin and bird plumage pictures. They reported a significantly larger EPN component for close-up snake skin pictures. In addition, in their second task, they presented participants with a set of partially exposed snake, spider and bird pictures. Likewise, partially exposed snakes triggered a higher EPN response compared to partially exposed spiders and birds. Further evidence that triangular snake-skin patterns in particular modulate the activity in human visual cortex has been provided by Grassini et al. (2019a).

The visual characteristics of snakes could be related to spatial frequencies. Snake skin scales and patterns represent a localized, high spatial frequency attribute and curvilinear shape a more global, low-spatial frequency attribute. The evidence on the importance of spatial frequency in threat detection is still ambiguous, although some studies have tried to shed light on the topic. Vuilleumier et al. (2003) argued that low-spatial frequency encoding of a threatening stimulus is enough to activate limbic structures (e.g., the amygdala) through subcortical pathways. Several behavioural studies are in support of this notion (Gao et al. 2017; Gomes et al. 2018). For instance, (Gomes et al. 2018) employing continuous flash suppression, explored the individual contribution of global and local features to rapid snake detection. They reported that in the original (no visual noise in the pictures) and the low-spatial frequency (LSF) conditions snakes were detected faster. This was not the case for the high-spatial frequency (HSF) condition. This infers that rapid snake detection could be mainly driven by global LSF attributes, such as the curvilinear shape of the animal. However, a “low-road” account has been criticized (De Cesarei and Codispoti 2013; Pessoa and Adolphs 2010) and several other behavioural studies have found opposing results that demonstrate that rapid fear detection relies on high rather than low-spatial frequencies (e.g., Stein et al. 2014).

It should be noted that Gomes et al. (2018) investigated the overall curvilinear shape of snakes and not the specific snake scale patterns. The contour of curvilinear snake shapes typically persist after low-pass filtering. Their results undoubtedly indicate that the curvilinear shape may be an important global (low frequency) visual characteristic for snake detection, but it does not definitely rule out the effects of high-frequency visual characteristics. As mentioned above, we have also found evidence that the curvilinear shape may play a role in threat detection (Van Strien et al. 2016). In our study, the EPN was significantly larger for snake than for worm pictures, indicating that the curvilinear shape is not the only factor causing the EPN snake effect and that probably other visual characteristics such as snake scale patterns may also play a role.

In a recent study, Beligiannis and Van Strien (2019) explored the role of high spatial frequency attributes in snakes. They presented participants with blurred (containing mainly low-spatial frequencies) and non-blurred (containing all spatial frequencies) pictures of snakes, spiders and birds and measured their EPN amplitudes. They reported that non-blurred snake pictures elicited a larger EPN compared to blurred snake pictures, suggesting that the EPN is larger when the snake skin patterns are clearly visible and supporting the notion that high spatial frequency local attributes play a role in snake detection. In their spatial frequency analysis, these authors found that non-blurred snake pictures (with all local characteristics, e.g., skin patterns and scales clearly visible) elicit excess high-frequency power compared to non-blurred spider and bird pictures. Likewise, Grassini et al. (2019b) found higher power in the higher spatial frequencies in snake and rope stimuli, before these authors equalized these stimuli for spatial frequency.

The visibility of snake characteristics triggering early attentional capture varies according to the relative distance (short or large) of the snake. This distance is a visual attribute that has not yet been studied with the RSVP paradigm; the proximity (perceived nearness) of the snakes in the pictures. As discussed above, early attentional capture leading to snake detection seems to be an automatic mechanism that was created and fine-tuned by evolutionary cues to promote primates’ survival. The mechanism’s role is to detect the snake as fast as possible for the primate to either avoid or keep enough distance from it. That makes its function relevant at larger distances as snakes pose a severe threat mainly at short distances, due to the snake’s short reactive distance (Soares et al. 2014). At short distances preventive detection has failed and belated detection occurs as part of a close encounter. It can, therefore, be hypothesized that pictures of snakes appearing at a short distance (high proximity) will elicit lower EPN amplitudes in the parietal occipital cluster compared to pictures of snakes appearing at a larger distance (low proximity). It is conceivable that the fast and automatic snake detection mechanism is less triggered by close encounters than by detection at larger distances. At larger distances, the snake detection mechanism is probably activated by the appropriate cues such as the curvilinear body shape and the skin patterns discussed above. At short distance encounters, these cues may be less visible, but also are less relevant, as preventive detection has failed. In addition to the lack of relevant cues needed for the activation of the snake detection mechanism, it could also be that during close encounters the primate is focusing on different visual characteristics of the snake, like the shape of its head, which is the most prominent feature in a close encounter. This belated detection would trigger autonomic responses, such as freeze or flight which are discussed in defense cascade models (Fanselow 1994; Wendt et al. 2017).

The present study explored whether the attribute of proximity (how close the stimulus appears) influences the early attentional capture mechanism in snake detection. To manage that, we employed close-up (high proximity, with the head of the animal clearly visible and in the foreground) and medium-shot (low proximity, full-body exposure) snake, spider and bird pictures. Because we have employed spider and bird pictures as control stimuli in our previous research, they were included in the present research as well. The selection of snakes and spiders as aversive stimuli is in line with previous research in which both snake and spider stimuli were found to be more readily associated with aversive unconditioned stimuli as compared to non-fear stimuli, such as flowers or mushrooms (Öhman and Soares 1998; Öhman and Mineka 2001), due to their supposed evolutionary “preparedness” (Mineka and Öhman 2002; Seligman 1971). Furthermore, birds have emerged to be reliable neutral (fear irrelevant) stimuli in RSVP research, as in many studies they exhibit the lowest arousal ratings and smallest EPN amplitudes as compared to snake and spider stimuli (e.g., Van Strien et al. 2014a, b; Beligiannis and Van Strien 2019; Van Strien and Isbell 2017). Based on the previous research, we expected higher EPN amplitudes in response to snake pictures when compared with spider and bird pictures. As explained above, we expected that medium-shot snake pictures would elicit higher EPN amplitudes than close-up snake pictures. Within the framework of the SDT, proximity may be less relevant for spider and bird detection.

In addition, to explore the extent to which high spatial frequency local and low-spatial frequency global visual features are visible in close-up versus medium-shot pictures, we conducted a spatial frequency analysis on both condition (medium shot, close-up) and category (snake, spider, bird). We expected that medium-shot pictures (particularly medium-shot snake pictures) would possess more power in the high spatial frequency bands than close-up pictures, as focal, high spatial frequency attributes would be better visible (e.g., scales and patterns on snake skin).

## Methods

### Participants

Thirty participants (15 males, 15 females, mean age = 20.1, range = 18–24 years) were recruited for this study. Participants were undergraduate students at Erasmus University and enrolled in the experiment in exchange for course credits. Signed informed consent was obtained at the start of the experimental session. The present research was part of a larger study in which this sample participated (see also Beligiannis and Van Strien 2019). The study was approved by the local ethics committee of the Department of Psychology, Education and Child Studies, Erasmus University Rotterdam.

### Fear questionnaires

Participants were asked to fill in two Fear Questionnaires, the Snake Phobia Questionnaire and the Spider Phobia Questionnaire. These were adopted versions of the Spider Phobia Questionnaire (Klorman et al. 1974; Muris and Merckelbach 1996). Each questionnaire contained 30 items where participants responded with “True” or “False” statements. Subjects were asked to complete the questionnaire by keeping in mind the expected amount of fear they would experience if they encountered one of these animals in real life and in their own personal space. The minimum score on both questionnaires was 0 and the maximum was 30. Participants completed this task before the RSVP task.

### Self-report measures

Participants had to rate on scales of 1–9 their valence and arousal ratings for the pictures used in the RSVP task. A computerized version of the Self-Assessment Manikin (SAM) questionnaire was used (Bradley and Lang 1994). Participants completed this task after the RSVP task.

### Stimuli and procedure

**Fig. 1:**
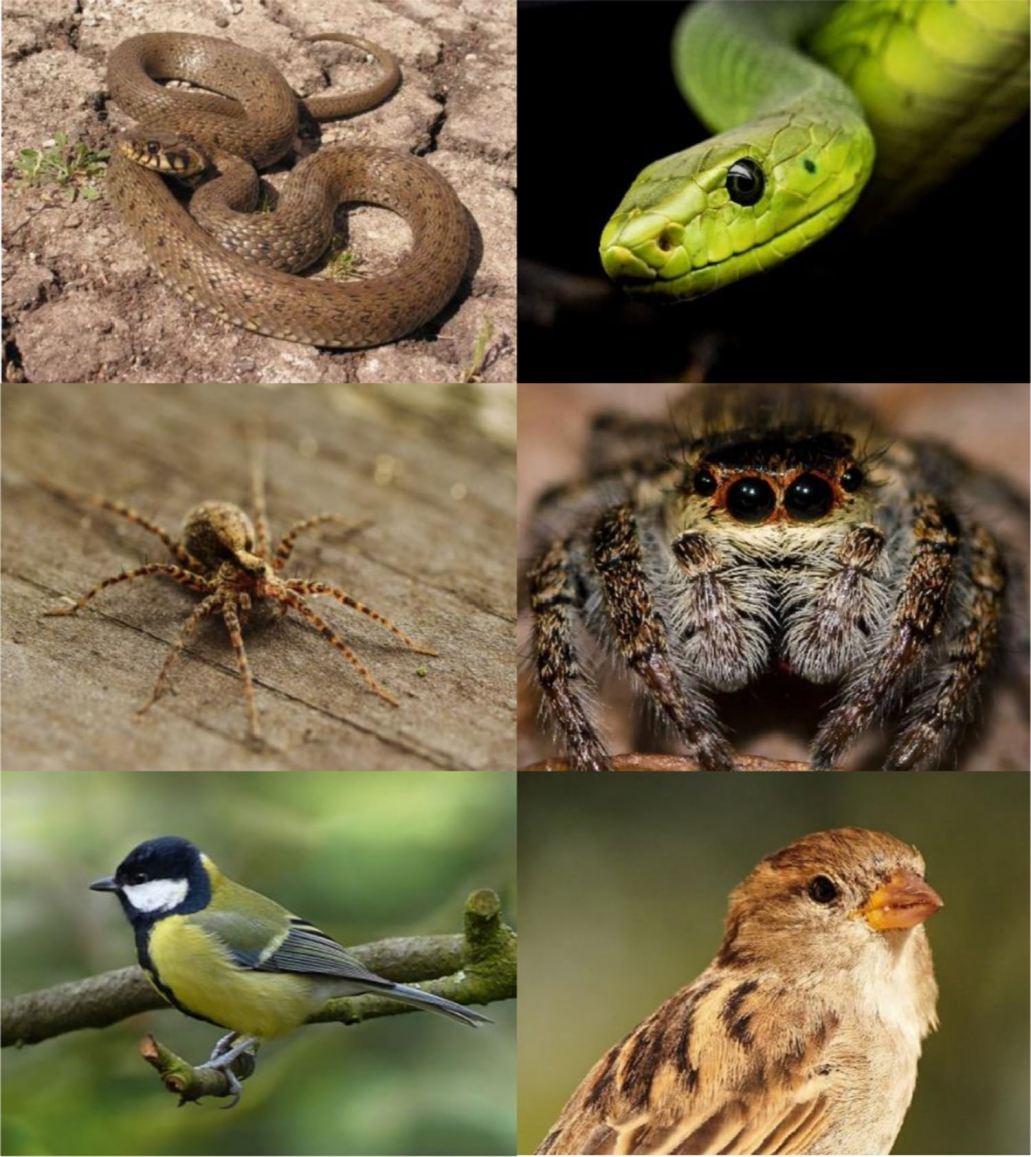
Examples of medium-shot (left column) and close-up (right column) snake, spider and bird pictures. For copyright reasons, the depicted photographs are public domain (pixabay.com); they are similar to the actual stimuli, but were not used in the experiment

Participants watched a RSVP of 600 snake, 600 spider and 600 bird pictures (3 categories, 2 conditions, 10 pictures per block, 30 repetitions per picture). All pictures were obtained from the internet. The medium-shot vs. close-up conditions were blocked and counterbalanced across participants, the snake, spider and bird categories were mixed and random within each block. Pictures had a size of approximately 600 × 450 and were displayed in a grey background. The medium-shot pictures showcased the animal in a “full-body” mode from a high angle. The close-up pictures exhibited full-head exposure, with the head being clearly visible in the center and on the foreground. The pictures were shown at a distance of 120 cm on a 20-inch PC monitor with a resolution of 1024 × 768 pixels. Examples of the medium-shot and close up stimulus categories are given in Fig. 1.

### EEG recording

A BioSemi Active-Two system was used to record brain activity during the RSVP task. The system used 32 pin type active Ag/AgCL electrodes on an elastic cap, following the rules of the international 10–20 system. Flat type active electrodes were used to record the Electro-ocular activity (EOG). For this purpose, two electrodes were placed above and below the left eye and another set of two electrodes on the outer corners of both eyes. Both EEG and EOG data were registered with a sampling rate of 512 Hz, 134 Hz low-pass filtering and 24-bit A/D conversion.

### ERP data analysis

ERP data were analyzed using Brain Vision Analyzer 2.0 (Brainproducts. Gilching, Germany). An average reference was used to re-reference all signals. Data were filtered phase-shift-free with a 0.10–30 Hz band pass (24 dB/Oct). We made corrections for existing ocular artifacts with the (Gratton et al. 1983). The ERP time window was set from 50 ms before and to 330 ms after stimulus onset. The ERP signals were baseline corrected relative to the mean of the 50 ms pre-stimulus period. We computed averaged ERPs for each combination of stimulus category (snakes, spiders, birds) and condition (medium shot, close-up). Epochs with a baseline to peak amplitude larger than ± 100 μV were omitted from the analysis. More than 99% of our time frames proved to be valid at the electrodes of interest for each stimulus category. The EPN data were measured at the parietal–occipital cluster (PO3, O1, Oz, O2 and PO4) and was defined as the mean activity at those electrodes between 225 and 300 ms upon stimulus onset.

### Spatial frequency analysis

The spectral compositions of the pictures that were used in the present research, were measured by employing a discrete wavelet analysis on each picture, using the Matlab routines *freqspat.m* and *freqspat_gui.m* as described and provided by Delplanque et al. (2007). With discrete wavelet analysis, the picture is decomposed in eight independent spatial frequency bands of which the power is determined. Small features (i.e. details) of a picture will result in higher power for high spatial frequencies, while large features will result in higher power for low-spatial frequencies. It should be noted that the spatial frequency analysis was done as a post hoc check and did not play a role in the picture selection.

### Luminance analysis

As our stimuli were not equated for luminance, we conducted a post hoc luminance analysis on the picture categories and conditions to examine possible luminance differences. We extracted the average RGB colour code compositions per picture using https://matkl.github.io/average-colour/. By applying the luminance function formula (0,2126*R + 0,7152*G + 0,0722*B), we calculated the luminance level for each picture.

### Statistical analyses

For the snake and spider fear scores, a repeated-measures ANOVA was employed with stimulus category (snake, spider) as factor. For the valence and arousal ratings, separate repeated measures ANOVAs were conducted with stimulus category (snake, spider, birds) and condition (medium shot, close-up) as factors. For the EPN, a repeated-measures analysis of variance (ANOVAs) was conducted with stimulus category (snake, spider, bird), condition (medium shot, close up) and electrode (PO3, O1, Oz, O2, PO4) as factors. These electrodes were selected as stimuli of evolutionary importance are believed to modulate the EPN amplitudes at the parietal–occipital cluster (Van Strien, Franken et al. 2014). Greenhouse–Geisser corrections were used when applicable. To compare the luminance levels of the pictures, a two-way between-groups ANOVA was employed with animal category (snake, spider, bird) and condition (medium-shot, close-up) as factors.

## Results

### EEG measures

**Fig. 2:**
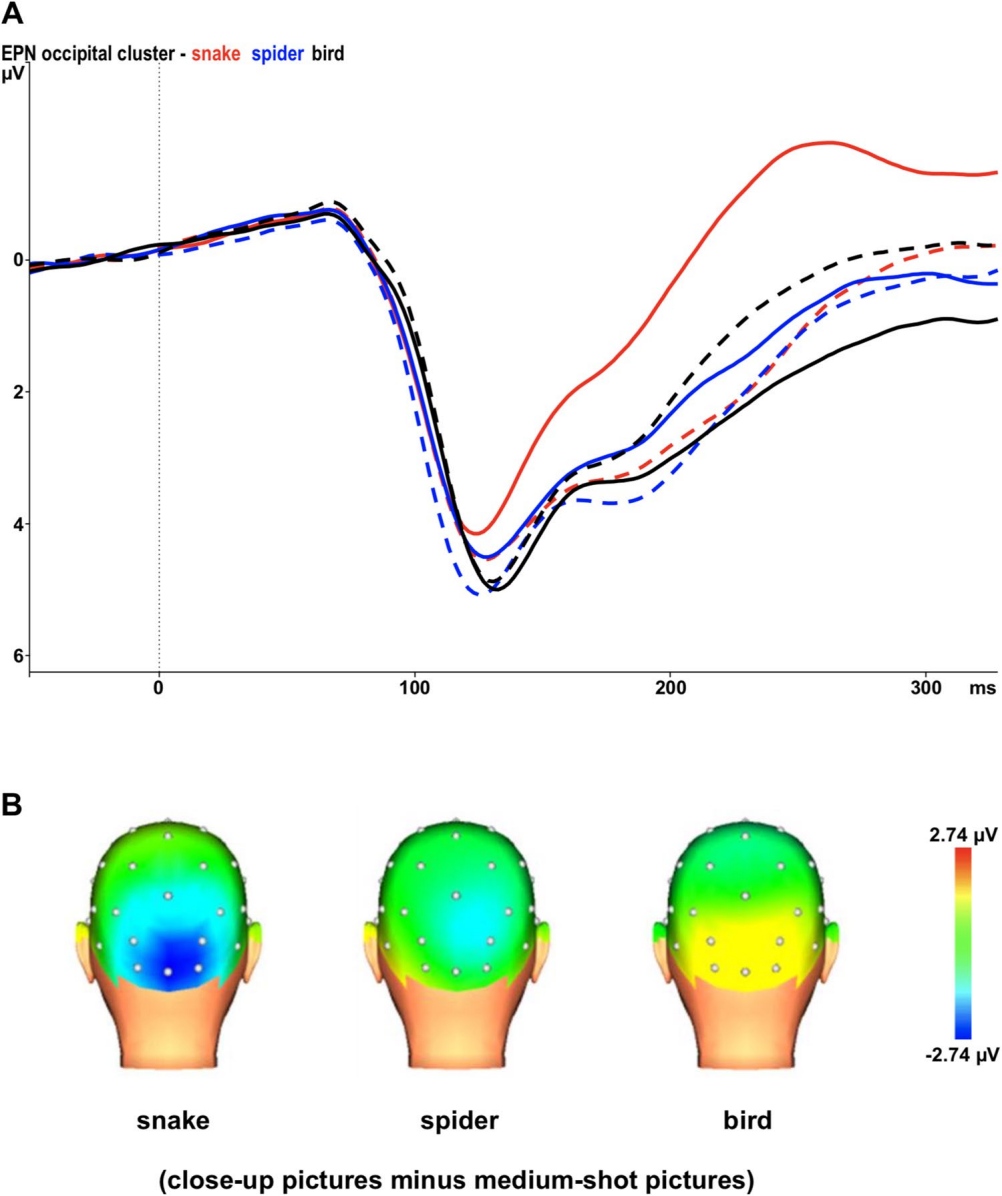
**a** Grand-average early posterior negativity (EPN) in response to medium-shot (solid lines) and close-up (dashed lines) snake, spider and bird pictures at the occipital cluster (O1/2, Oz, PO3/4). Negativity is up. **b** Topographic maps of the differences in EPN mean amplitudes (225–300 ms) between medium-shot vs. close-up snake, spider and bird pictures

Figure 2a displays the EPN in response to medium and close-up snake, spider and bird pictures at the occipital cluster. We found an overall significant main effect for category, *F*(2,58) = 34.46, *ε* = 0.837, *p* < 0.001. Pairwise comparisons using Bonferroni correction showed that snake pictures generated a significantly more negative EPN than spider (*p* < 0.001) and bird pictures (*p* < 0.001). The ANOVA also indicated a significant main effect for condition, *F*(1,29) = 7.16, *p* = 0.012, suggesting significantly larger EPN amplitudes for medium-shot than close-up pictures. These main effects were qualified by a significant interaction of category and condition *F*(2,58) = 131.72, *ε* = 0.893, *p* < 0.001. Medium-shot snake pictures elicited higher EPN amplitudes as compared to close-up snake pictures (*p* < 0.001) and close-up bird pictures elicited higher EPN amplitudes as compared to medium-shot bird pictures (*p* < 0.001). EPN responses to spider pictures did not significantly vary per condition.

The interaction of condition and electrode *F*(4,116) = 5.21, *ε* = 0.741, *p* < 0.001 and the interaction of category and electrode *F*(8,232) = 11.67, *ε* = 0.594, *p* < 0.001, were also significant. Figure 2b displays the topographical differences between close-up and medium-shot snake, spider and bird pictures.

### Fear measures

Spiders were scored as being slightly more fearful (*M* = 9.77, *SD* = 7.01, range 2—27) than snakes (*M* = 9.63, *SD* = 6.09, range 1—21). The difference between snake and spider fear scores was not statistically significant, *F*(1,29) = 0.011, *p* = 0.916.

### Valence and arousal ratings

The mean valence and arousal ratings for snake, spider and bird pictures are given in Table 1.

**Table 1:** Participants’ mean arousal and valence ratings (and standard deviations)

Stimulus category	Valence (SD)	Arousal (SD)
Medium-shot snake	3.95 (1.25)	4.22 (2.11)
Close-up snake	4.35 (1.66)	4.07 (2.11)
Medium-shot spider	3.21 (1.28)	4.86 (2.22)
Close-up spider	3.40 (1.69)	5.47 (2.27)
Medium-shot bird	6.41 (1.28)	1.36 (.61)
Close-up bird	6.32 (1.14)	1.56 (.66)

Valence and arousal ratings are based on a rating scale from 1 to 9

There were significant main effects of category for both arousal, *F*(2,58) = 56.22, *ε* = 0.974.

*p* < 0.001 and valence ratings, *F(*2,58) = 46.03, *ε* = 0.793, *p* < . 001. Snake and spider pictures yielded higher arousal ratings compared to bird pictures (both *p*-values < 0.001), while spider pictures yielded higher arousal ratings than snake pictures (*p* = 0.014). Bird pictures elicited significantly higher valence ratings as compared to snake and spider pictures (both *p* values < 0.001). While snake pictures elicited higher valence ratings than spider pictures (*p* = 0.003) The effect of condition was statistically significant only for arousal, *F(*1,29) = 6.32, *p* = 0.018, with close-up pictures eliciting significantly higher arousal ratings than medium-shot pictures.

The interactions between category and condition were significant for both arousal, *F(*2,58) = 7.49, *ε* = *0.7*78, *p* = 0.003 and valence, *F(*2,58) = 3.74, *ε* = *0.8*83, *p* = 0.035. More specifically close-up spider pictures exhibited higher arousal than medium-shot spider pictures and close-up bird pictures exhibited higher arousal than medium-shot bird pictures (both *p* values ≤ 0.015). Close-up snake pictures exhibited higher valence ratings compared to medium-shot snake pictures (*p* = 0.031).

### Spatial frequency analysis

**Fig. 3:**
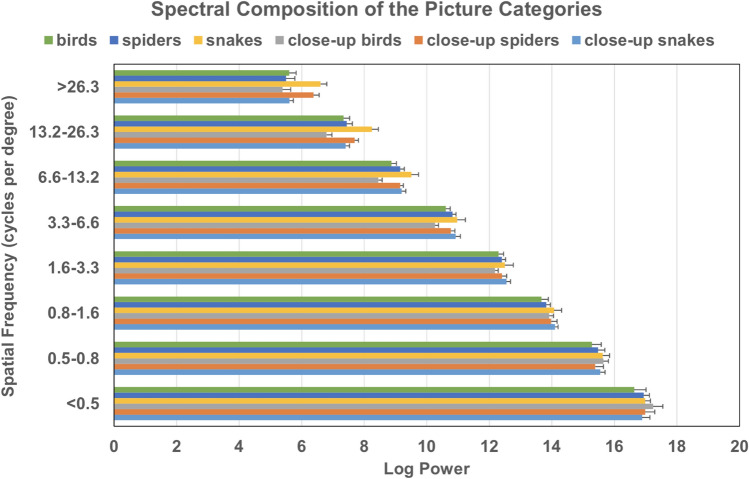
Mean energy for each frequency band as a function of picture category and condition. Error bars depict standard error of means. Frequency bands are expressed in cycles per degree of visual angle. High spatial frequencies are on top

From Fig. 3, it can be seen that medium-shot and close-up pictures exhibited differences in energy in the higher frequency bands in particular. Mann–Whitney tests for each category and frequency band revealed significantly lower energy for close-up as compared to medium-shot snake pictures in the two highest spatial frequency bands (> 26.3 cycle/degree, *p* = 0.002; 13.2–26.3 cycle/degree, *p* = 0.007). Energy was significantly higher for close-up as compared to medium-shot spider pictures in the highest frequency band (> 26.3 cycle/degree, *p* = 0.034). Finally, energy was significantly lower for close-up as compared to medium-shot bird pictures in the 13.2–26.3 cycle/degree frequency band (*p* = 0.041) and the 3.3–6.6 cycle/degree frequency band (*p* = 0.049).

### Luminance analysis

The ANOVA indicated that there were no significant main effects for picture category, *F(*2,54) = 0.27, *p* = 0.763 and condition *F(*1,54) = 0.01, *p* = 0.953, nor a significant interaction, F(2,54) = 0.50, p = 0.607, suggesting that there were no significant differences in luminance levels between the picture stimuli.

## Discussion

To explore the effects of the depicted proximity of snakes on the EPN, we presented participants with a RSVP paradigm with full-animal body exposure (medium-shot) and partial animal exposure (close-up, head fully visible) pictures, while recording their EEG activity. The EPN corresponds to early attentional capture and is enhanced by evolutionary relevant stimuli (Schupp et al. 2003). We hypothesized that the EPN would be larger for snake pictures compared to spider and bird pictures and that the EPN would be larger for medium-shot than close-up snake pictures.

Overall, in line with the previous research, snake pictures elicited the highest EPN amplitudes compared to the other animal categories across both picture conditions (medium-shot and close-up). This provides further support for SDT along with previous research (He et al. 2014; Grassini et al. 2016; Van Strien et al. 2014a, b; Van Strien et al. 2014; Van Strien and Isbell 2017). More specifically, medium-shot snake pictures (low proximity) elicited significantly higher EPN amplitudes compared to close-up snake pictures. The outcome that proximity modulates the EPN snake effect, acts as further support for the idea that the availability of local (high frequency elements, such as scales and patterns which are present in medium-shot pictures) or global (e.g., the curvilinear shape) visual attributes of the snake, drive early attentional capture. It is interesting that exposure to pictures of snake skin or partial snake bodies clearly elicits the EPN snake effect (Van Strien and Isbell 2017), whereas exposure to close-up pictures of snake heads does not. It may be that that the fast and automatic snake detection mechanism, as reflected by the EPN, is less triggered by a close encounter with snakes than by the presence of snakes at larger distances. For the detection at larger distances, cues such as the curvilinear body shape and the skin patterns may play an important role. At short distance encounters however, detection is obvious and probably more urgent defense mechanisms will be activated.

We conducted a spatial frequency analysis to explore whether medium-shot snake pictures do hold more high spatial frequency power compared to close-up snake pictures. The analysis indicated that medium-shot snake pictures (containing local high spatial frequency elements), indeed possessed significantly more power in the two highest spatial frequency bands than close-up snake pictures. This indicates that high spatial frequency features in full-body exposure (medium-shot) snake pictures may play a role in snake detection. The snake skin’s scales and patterns may constitute such local features and we do expect them to be reflected in high spatial frequencies, but that may be dependent on the characteristics of the stimuli employed. Spatial frequency analyses from the previous research seem to support this claim (Beligiannis and Van Strien 2019; Grassini et al. 2019b).

Since our picture stimuli were not equated for luminance, we conducted a post hoc luminance analysis to compare the luminance levels of the pictures. We did not find any significant category or condition differences, suggesting that the pictures contain similar luminance levels. It should be noted that, concerning low level features, previous research with brightness equated grayscale pictures (He et al. 2014) or with contrast and luminance equated colour pictures (Grassini et al. 2016) also have yielded distinct EPN snake effects.

In this study, we considered snakes as one uniform animal category. The recent studies have suggested that subdivisions, such as venomous versus non-venomous snakes, snakes with versus without triangular patterns or fear-inducing versus disgust-inducing snakes may have different effects on both human fear and electrophysiological responses (Grassini et al. 2019a; Rádlová et al. 2020). Our future research will examine to what extent individual pictures of different kinds of snakes modulate the EPN amplitude.

The present results demonstrate a role of spatial frequencies in early automatic visual attention. Previous research has indicated that rapid threat detection depends on the availability of high spatial frequency attributes (Stein et al. 2014). That is in line with our present findings, as such attributes were available in the medium-shot (low proximity) pictures and resulted in an elevated EPN as compared to close-up (high proximity) snake pictures.

Surprisingly, close-up bird pictures elicited significantly higher EPN amplitudes compared to medium-shot bird pictures. This suggests that close-up bird pictures have a higher attentional value than medium-shot bird pictures, but we can only speculate why this might be the case. It may be worthwhile to further investigate this outcome, for instance by examining the EPN responses to close-up and medium-shot pictures of other animals, such as rodents or dogs.

The participants’ valence and arousal ratings for the three picture categories were comparable with the previous research, as were the similar snake and spider fear scores (Beligiannis and Van Strien 2019; Van Strien et al. 2014a, b). The arousal ratings indicated that close-up pictures of both birds and spiders were more arousing than the medium shots, whereas there was no condition effect for snake pictures. Close-up spider pictures in particular show much details that may go unnoticed in medium shots and this may thus invoke a higher sense of arousal. Valence ratings were higher for close-up versus medium-shot snake pictures. It is not exactly clear why, but it could be that the valence ratings to spider pictures are related to some counterintuitive attraction to these animals. The ratings for snake and spider fear were comparable and were relatively low. The Fear Questionnaires were administered prior to the RSVP procedure, which might have influenced attention to snakes and spiders in the subsequent experimental run. However, as the average fear scores for snakes and spiders were comparably low, this most probably will not have affected the current EPN results.

The present study did not provide a definite answer on the relative distance needed to activate the snake detection mechanism, as we only explored two conditions; medium-shot (low proximity) and close-up (high proximity). Future research may further clarify the role of proximity on the snake EPN effect, by employing different variations in proximity (i.e., various distances).

To conclude, we found significantly enhanced EPN amplitudes in response to snake pictures as compared to spider and bird pictures. The snake EPN effect was significantly larger for medium-shot pictures (full-body exposure) as compared to close-up (mainly head exposure) pictures. This suggests that medium-shot pictures may contain local attributes (patterns, scales on snake skin) that are vital for rapid snake detection. The spatial frequency analysis conducted on the pictures indicated that medium-shot pictures contain more high spatial frequencies as compared to close-up pictures. We conclude that the proximity of the animal in the picture may be a relevant feature for faster snake detection at larger distances; however, its role might be mediated by the spatial frequency characteristics of the picture.
